# Acetylcholine and synaptic homeostasis

**DOI:** 10.1186/1471-2202-13-S1-O6

**Published:** 2012-07-16

**Authors:** Christian G Fink, Victoria Booth, Michal Zochowski

**Affiliations:** 1 Department of Physics, University of Michigan, Ann Arbor, MI 48104, USA; 2 Departments of Mathematics and Anesthesiology, University of Michigan, Ann Arbor, MI 48104, USA; 3 Biophysics Program, University of Michigan, Ann Arbor, MI 48104, USA

## 

The synaptic renormalization hypothesis posits that a primary function of sleep is to maintain synaptic homeostasis [1]. According to this theory, the flood of sensory signals processed by the brain during waking results in global potentiation of cortical synapses, a process which consumes energy and space and therefore cannot continue unabated. Sleep is therefore a period of global synaptic downscaling that maintains homeostasis, thereby conserving energy and cortical space. Specifically, it is slow-wave activity (SWA) during NREM sleep that is thought to induce this depotentiation. While evidence in support of both global potentiation of synapses during waking [2] and SWA-mediated downscaling of synapses during sleep [3] continues to mount, there is still much uncertainty about the biophysical mechanisms which may contribute to either synaptic upscaling or downscaling [4].

**Figure 1:**
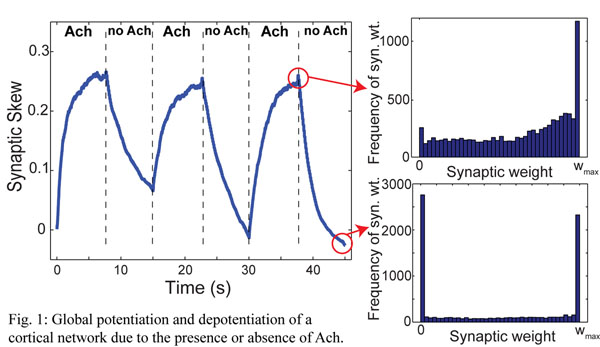


Waking and sleep states are promoted by the activity of brainstem and hypothalamic neuronal nuclei that express key neurotransmitters in thalamic and cortical brain regions [5]. Waking is characterized by high levels of noradrenaline, serotonin, histamine and acetylcholine, while all these neurotransmitters are at low levels during NREM sleep. We propose that the influence of acetylcholine (ACh) may provide a mechanism for both upscaling and downscaling of cortical synapses. Specifically, experimental studies have shown that ACh modulation switches the phase response curves of cortical pyramidal cells from Type II to Type I. Our computational studies of cortical networks show that the presence of ACh induces cellular and network dynamics which lead to net synaptic potentiation under a standard STDP rule, while the absence of ACh alters dynamics in such a way that the same STDP rule leads to net depotentiation (see Fig. 1). Thus the well-established prevalence of ACh in cortical circuits during waking may lead to global synaptic potentiation, while the absence of ACh during NREM sleep may lead to global depotentation.

Counter-intuitively, the global potentiation induced by the presence of ACh in our simulated networks is due to asynchronous activity. This is due to the fact that in the asynchronous state, there exists important statistical structure to the network dynamics, so that post-synaptic neurons are more likely to fire immediately after (rather than before) a pre-synaptic action potential, thus leading to net potentiation of the network due to STDP.
